# Nanogels with Selective Intracellular Reactivity for Intracellular Tracking and Delivery

**DOI:** 10.1002/chem.202001802

**Published:** 2020-10-19

**Authors:** Guangyue Zu, Olga Mergel, Laís Ribovski, Reinier Bron, Inge S. Zuhorn, Patrick van Rijn

**Affiliations:** ^1^ University of Groningen University Medical Center Groningen Biomedical Engineering A. Deusinglaan 1 9713 AV Groningen The Netherlands

**Keywords:** controlled release, delivery, hydrolysis, multimodal, nanogel

## Abstract

A multimodal approach for hydrogel‐based nanoparticles was developed to selectively allow molecular conjugated species to either be released inside the cell or remain connected to the polymer network. Using the intrinsic difference in reactivity between esters and amides, nanogels with an amide‐conjugated dye could be tracked intracellularly localizing next to the nucleus, while ester‐conjugation allowed for liberation of the molecular species from the hydrogel network inside the cell, enabling delivery throughout the cytoplasm. The release was a result of particle exposure to the intracellular environment. The conjugation approach and polymer network building rely on the same chemistry and provide a diverse range of possibilities to be used in nanomedicine and theranostic approaches.

## Graphical abstract

**Nanogels** are highly versatile hydrogel nanoparticles that have much potential in nanomedicine and theranostics. The key issue remains to control the location of release of active conjugated moieties and controlling when and if conjugated structures will be liberated. Here, a versatile approach is presented that allows selective intracellular release of small molecules, which would tremendously affect the field of nanomedicine.

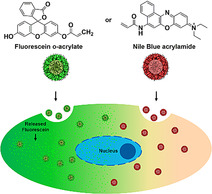

Within the field of nanomedicine, delivery vehicles are regarded as one of the major strategies to successfully protect and deliver pharmaceutical components.^[1]^ However, it also becomes more apparent that different delivery strategies are needed for different diseases and different cell types.^[2]^ In drug delivery, both the release mechanism and the body distribution of the vehicle play an important role. Theranostic approaches, which combine diagnosis and treatment of disease, serve to visualize drug delivery vehicles.[^2^, ^3^, ^4^] So far, many of the theranostic approaches are performed with solid nanoparticles (inorganic/organic) or liposomes/polymersomes.[^2^, ^3^, ^4^, ^5^] A major advantage of liposomes and polymersomes is that these allow for the use of the complete particle volume for drug loading, while for most of the solid particles only the particle surface is available. However, solid structures have generally an enhanced stability towards different (bio)chemical environments, solvents, and mechanical stress.

Hydrogel nanoparticles fulfill an important niche by combining high drug loading capacity with tunable stability and resilience. They consist of a covalent polymer network which provides the stability to cope with chemical environments and solvent compositions, and can be designed to respond to various stimuli, such as temperature, pH, and light.^[6]^ On top of that, hydrogel nanoparticles can undergo high deformations without breaking.^[7]^

Hydrogel nanoparticles or nanogels, have been used in various fields of research including catalysis,[^8^, ^9^] selective diagnostics and delivery,[^10^, ^11^] and anti‐fouling coatings.[^12^, ^13^] Even though these nanogels have found their way into the biomedical field,[^14^, ^15^] theranostics and multimodalities remain underdeveloped and mostly hybrid structures are being used (inorganic/organic).[^16^, ^17^, ^18^] Few examples have been developed that rely solely on hydrogel structures[^17^, ^19^, ^20^, ^21^, ^22^, ^23^] and when doing so, the multimodality originates from a single molecular species,^[24]^ which limits the general applicability. Therefore, creating a more generalized approach based on the same chemistry but with the possibility of easy exchanging the active structure provides a powerful approach and further develop the field of multimodal nanogels in nanomedicine.

Our aim was to utilize the intrinsic difference in reactivity between ester‐ and amide‐conjugation to tune the susceptibility of nanogel hydrolysis towards the intracellular environment and thereby controlling selective release of incorporated molecular structures. Esterases, found in the intracellular environment, are known to have chemical selectivity.^[25]^ The modalities with different intracellular stability were incorporated without altering the overall development of the nanogel and using acrylic esters, release of incorporated molecules was triggered within the cell while outside the cell release did not occur. Using acrylamide conjugation, release was absent both outside and inside the cell, and particle tracking was facilitated. Intracellular stability of the nanogels was corroborated by the response of the nanogels toward cell lysate, which triggered similar release profiles. The presented versatile and innovative approach will trigger development of new multimodal particles for drug delivery and theranostics and exemplifies easy diversification using simple chemical approaches and introducing these within the same particle without affecting their function.

The nanogel formation is based on well‐established precipitation polymerization (Scheme 1).^[25]^ The initiation of a free radical polymerization leads to small oligomers that have different solubility than the used monomers under set conditions, which leads to the precipitation and formation of small colloids. These colloids will grow in size and after termination of the polymerization reaction and reestablishment of solubilizing conditions of the polymers, the network within the colloids will be rehydrated, resulting in a nanogel. By using a mixture of monomers, various functionalities can be introduced such as degradability, responsiveness (temperature, pH, light, redox reactions), simply by adding other monomers that will take part in the polymerization process.^[25]^ Here, fluorescein‐acrylate (FL‐Ac) and Nile blue acrylamide (NB‐AAm) have been used to demonstrate the principle of using the specific intracellular environment to selectively release or retain components in the nanogel, respectively (Figure S1). *N*‐Isopropylmethacrylamide (NIPMAM) is used as the responsive unit that facilitates the use of precipitation polymerization. The volume phase transition temperature (VPTT) of NIPMAM is around 45 °C and above this temperature, dehydration occurs followed by precipitation and colloid formation. This process can be repeated, which will provide a shell with a different composition around the initially formed core particle.[^26^, ^27^] After the formation of the FL and NB‐labeled core particles, a shell consisting of NIPMAM, BIS, and 3‐acrylamidophenylboronic acid (APBA) was formed (Scheme 1) which was evaluated by ^1^H NMR spectroscopy (Figure S2). It is know that APBA is enhancing cellular uptake in cancer cells, due to its ability to bind to the overexpressed sialic acid on the surface of cancer cells.[^28^, ^29^, ^30^] The difference in extracellular and intracellular reactivity as well as the intrinsic stability difference between esters and amides facilitate control over the release of incorporated molecules from the nanogel at the desired location (intracellular). After uptake of nanogels by cells via endocytosis, it was envisioned that there would be a fast release of ester‐conjugated structures from the nanogel due to the presence of esterases in endosomes, whereas the amide‐conjugated structures will remain associated with the nanogel as illustrated in Scheme 1 and Figure S1.

**Scheme 1:**
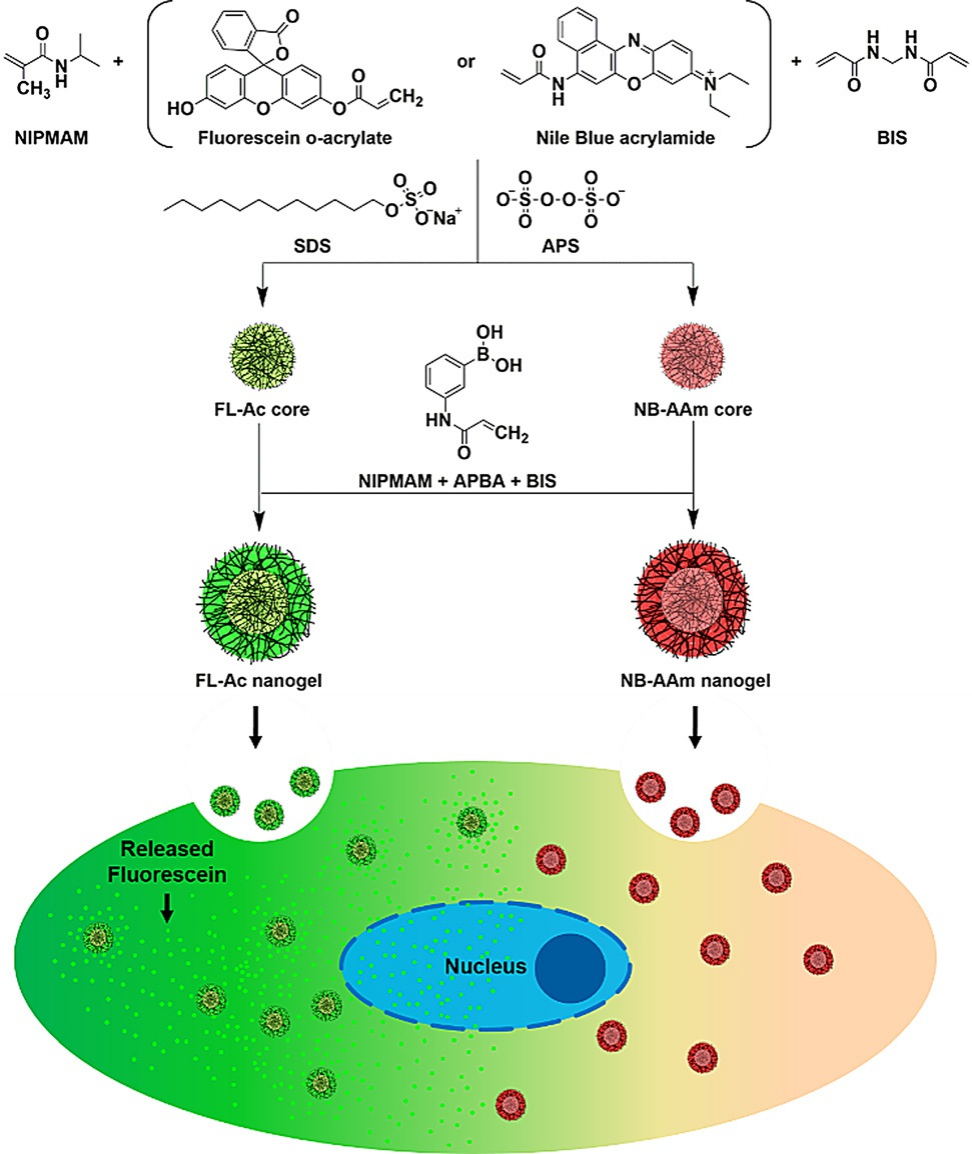
Synthetic approach for the multimodal nanogels through controlled precipitation polymerization in a two‐step fashion to create the core and subsequently the cell recognizing shell. Below, a schematic representation of the overall behavior of release and tracking outside and within the cell.

The nanogels used are primarily based on NIPMAM, which will dehydrate upon increasing the temperature to above the VPTT of 45 °C, which induces a decrease in size as shown by the temperature dependent dynamic light scattering (DLS) measurements (Figure S3 A). The VPTT is far above 37 °C, that is, body temperature, at which cell cultures are primarily done. Clearly, similar handling and incubation temperatures will apply in a clinical setting. At these conditions, temperature‐induced changes in the nanogels are not desired, as our aim is to elucidate the release profiles of the nanogels in response to the intracellular environment. Additionally, a collapsed matrix of the nanogel would inhibit proper exposure of the FL‐Ac nanogel to the intracellular esterase and could inhibit the hydrolysis. At room temperature (20 °C) the hydrodynamic diameter for NB‐AAm nanogel was 460 nm while at 37 °C the diameter reduced slightly to 430 nm. For the FL‐Ac nanogel the difference in hydrodynamic diameter was negligible, both around 300 nm (Figure S3 A). The diameter of both nanogels decreased upon increasing the temperature far above 37 °C and is therefore not considered to have any influence on cellular uptake or release events. The transmission electron microscopy (TEM) images confirm the presence of particle structures (Figure S3 B,C). The diameters were approximately 320 nm for the NB‐AAm nanogel and 200 nm for FL‐Ac nanogel, which are slightly smaller than the hydrodynamic diameters in the swollen state observed by using DLS as the nanogels are in dry state when measured with TEM.

It was envisioned that the intrinsic difference in reactivity of ester‐ and amide‐conjugated structures could be utilized to control the release profile of nanogels following their exposure to the intracellular environment. Intracellular esterases are capable of hydrolyzing a variety of substrates, while amidases are more selective toward specific peptide bonds.^[31]^ After nanogel internalization by cells, the difference in enzymatic susceptibility is utilized to initiate a release of ester‐conjugated structures, here a fluorescein‐ester conjugated moiety, whereas amide‐conjugated structures, here a Nile blue‐amide conjugated moiety, remain confined to the nanogel.

Confocal laser scanning microscopy (CLSM) of MCF‐7 breast cancer cells incubated with NB‐AAm nanogel for 2 h revealed a punctate fluorescent pattern in the cell cytosol, suggesting the presence of nanogels in endosomes (Figure 1 A). Incubation of MCF‐7 cells with FL‐Ac nanogel showed in addition to fluorescent spots, a diffuse cytosolic fluorescence. In addition, reticular structures were visible next to the nucleus, reminiscent of endoplasmic reticulum (Figure 1 A). Clearly, the two types of nanogels displayed distinct intracellular distributions following their incubation with MCF‐7 cells. To exclude that the intracellular distribution differences were a result of differences in nanogel cytotoxicity, a cell viability assay was performed. Both types of nanogels were identified to be non‐toxic at the concentrations used within the experiments (Figure S4). The internalization of the nanogels rather than being confined to the surface of the cells was confirmed analyzing the fluorescence signal in the *z*‐direction (Figure S5), which shows that the fluorescent signal is present within the cells.

**Figure 1:**
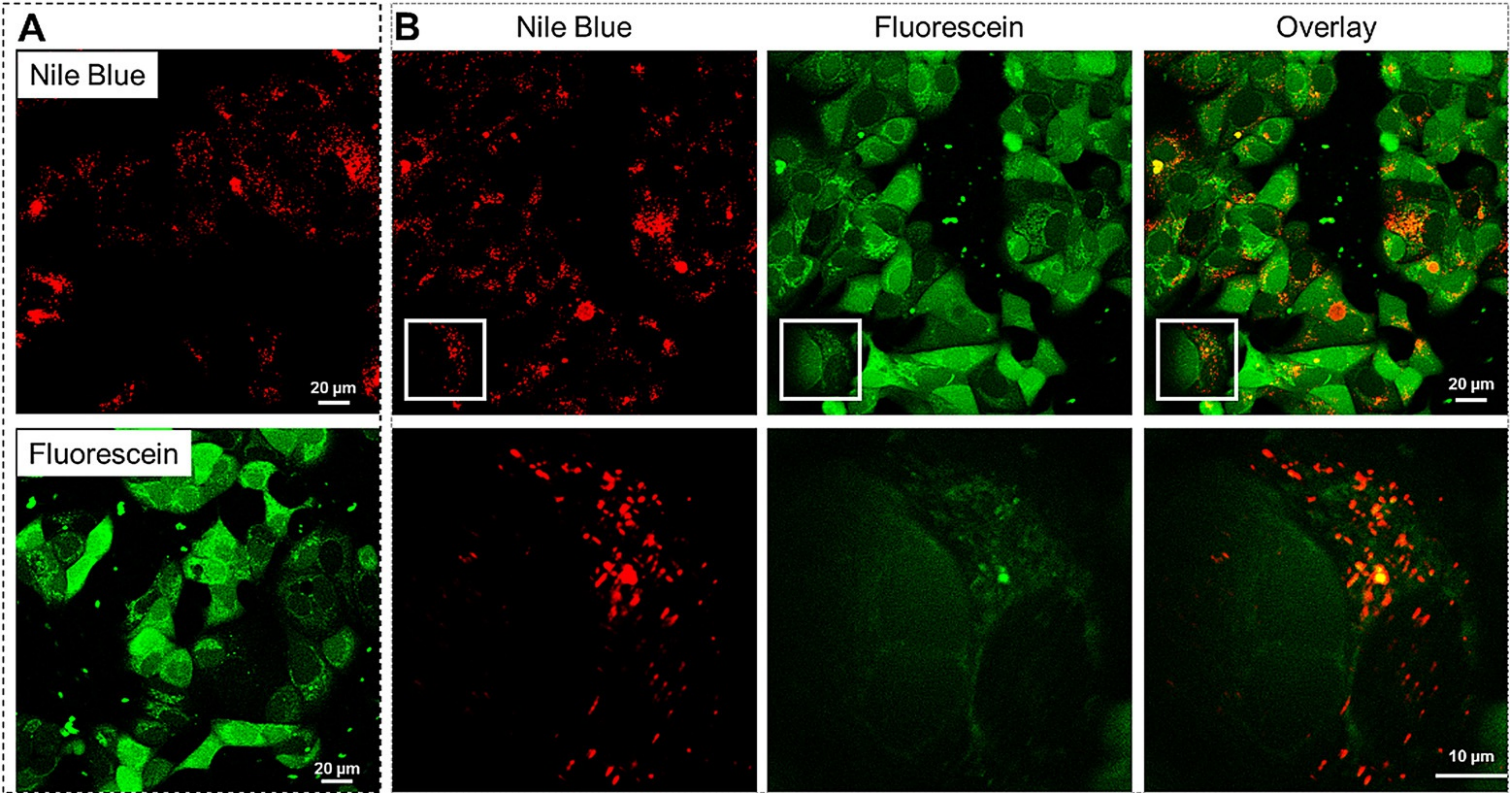
Representative confocal images of MCF‐7 breast cancer cells treated with (A) FL‐Ac nanogel and NB‐AAm nanogel, separately, (B) a mixture of FL‐Ac and NB‐AAm nanogels, for 2 h. Nile blue (red) is present as distinct dots within the cell cytosol. Fluorescein (green) mainly shows a diffuse cytosolic localization. In addition, it is visible as reticular structures next to the nucleus, reminiscent of endoplasmic reticulum. Amplified images (bottom row) further demonstrate that the ester‐conjugated fluorescein (green) shows partial overlap with the distinct dots presented by the amide‐conjugated Nile blue (red). In addition, the ester‐conjugated fluorescein (green) shows a diffuse pattern throughout the cell.

Next, MCF‐7 cells were incubated with a mixture of NB‐AAm and FL‐Ac nanogels. Figure 1 B shows that both nanogels were efficiently internalized by the cells, to a similar extent as upon separate incubations with the nanogels. Furthermore, MCF‐7 cells showed a partial overlap of fluorescein spots with Nile blue punctate in the cell cytosol, which most likely indicates colocalization of the fluorescent nanogels within endosomal structures (Figure 1 B and Figure S6). In addition, the fluorescein label was widely distributed over the entire cell. This highly diffuse pattern of the FL‐Ac label indicates that the dye was efficiently cleaved from the nanogel, most likely due to the presence of endosomal esterases, and released from endosomes. Typically, esterases have a broader range of substrate acceptance for hydrolysis than amidases. Therefore, the NB‐AAm label remained associated with the nanogel and confined to endosomes. Herewith, we show a simple approach to trigger intracellular cargo release or enable cargo confinement, by means of an intrinsic difference in reactivity of build‐in linkages inside the nanogel network.

While the selectivity was illustrated using a mixture of the nanogels, for theranostic and/or dual drug delivery approaches, both properties should be incorporated into the same nanogel without affecting the individual characteristics. Therefore, a nanogel was prepared containing both the ester‐ and amide‐conjugated dyes and the same intracellular release study was performed, which also excludes the possibility that the differences in fluorescence patterns observed for the two types of nanogels are caused by differences in their size (Figure S3). Figure 2 shows the same distinct patterns for fluorescein and Nile blue as was observed from mixing the two nanogels, only now both originate from the same nanogel. The fluorescence signal from the ester‐conjugated fluorescein is found both punctuated and cytosolic while the signal from the amide‐conjugated Nile blue is exclusively punctuated. This conceptual approach illustrates the delivery potential combined with traceability of such nanogels.

**Figure 2:**
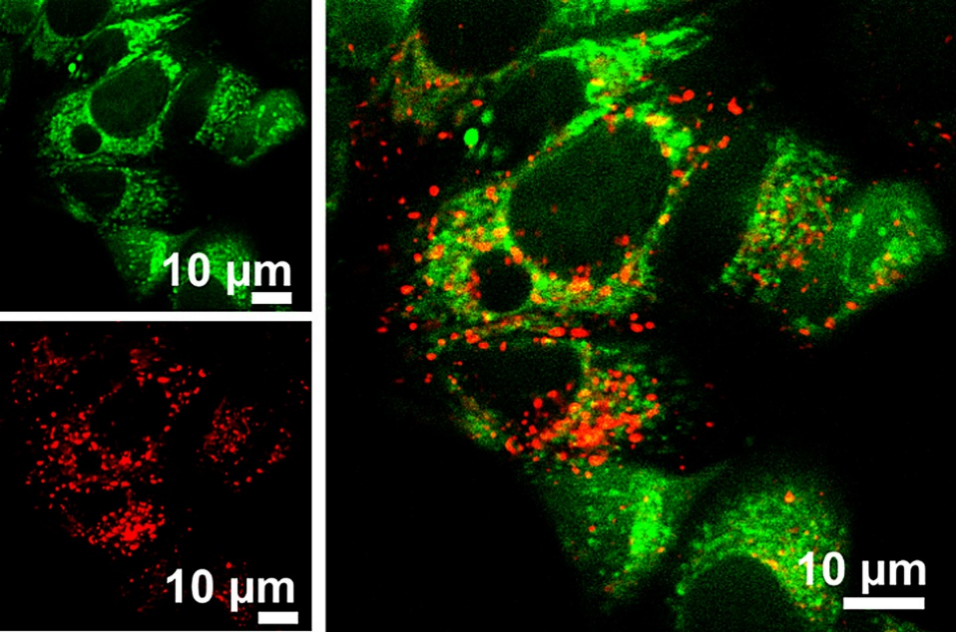
Representative confocal image of MCF‐7 breast cancer cells treated with Fl‐NB‐nanogels for 2 h. Nile blue (red) is present as distinct dots within the cell cytosol while fluorescein (green) is present as dots and shows a diffuse distribution in the cell cytosol.

Uptake of nanoparticles by cells primarily occurs via endocytosis. If nanogels are internalized via the endosomal pathway, then hydrolysis could also be facilitated by the lowering in pH. It is well known that the pH within an endosome decreases to pH value of 6.5 for early endosomes and continues to be lowered to 5.5 for late endosomes and 4.5 for lysosomes.^[32]^ This lowering of pH enhances the hydrolysis of esters. In order to identify if FL‐Ac release from the nanogels is dependent on a drop in (endosomal) pH, we exposed the different nanogels to cell lysate at pH≈7. The cell lysate contains all enzymes found intracellularly, without the need of going through the uptake process. Thereby, the pH shifts at the different endosomal stages are omitted. After exposure of the nanogels to cell lysate and subsequently removing the nanogels via ultracentrifugation (Figure S7), the fluorescence signal of the supernatant was determined in order to analyze the release of fluorescent cargo from the nanogels. Fluorescence that was released from nanogels exposed to water served as a control. Figure S7 shows the absence of fluorescence signal in the supernatant of NB‐AAm nanogels exposed to cell lysate, which was identical to that of the nanogel not exposed to cell lysate. In sharp contrast, the FL‐Ac nanogel supernatant did not show fluorescence signal after exposure to water (Figure 3), while in the presence of HBSS buffer a small fluorescence signal was detected. However, when in presence of cell lysate, the fluorescence intensity at the maximum wavelength (*λ*_max_=518 nm) is 13 times higher as compared to HBSS buffer alone, showing the substantial influence of the intracellular environment on the release. As the pH within these experiments was maintained at ≈7, we can conclude that cell lysate components, presumably esterases, are able to selectively hydrolyze the FL‐Ac conjugates. These data were supported by bafilomycin A1 inhibition experiments. MCF‐7 cells were treated with nanogels in the absence and presence of bafilomycin A1, which inhibits the acidification of endosomes. As shown in Figure S8, the FL‐Ac nanogel displayed the same release pattern in the presence of bafilomycin A1 as in its absence, indicating that the FL‐Ac can be released from the nanogel and redistributed throughout the cell without exposure to low pH.

**Figure 3:**
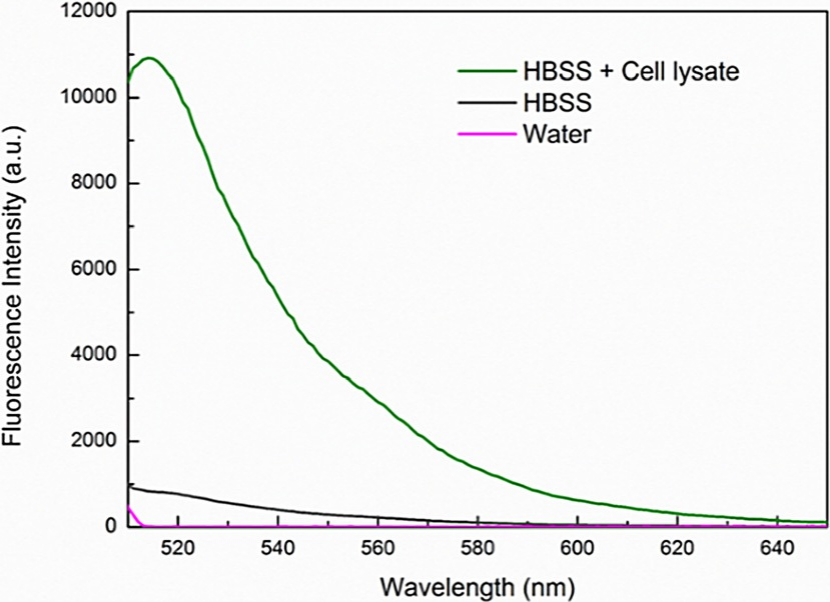
Fluorescence emission spectra of the supernatants of the ester‐conjugated (FL‐Ac) nanogels after exposure to water, HBSS buffer, and HBSS buffer with cell lysate and subsequent removal of the nanogels by centrifugation to illustrate the release properties specific to the intracellular environment.

We show that the difference in intracellular reactivity between esters and amides provides a powerful tool to decide between intracellular cargo release and confinement in nanogels. Controlled release may serve to mediate drug delivery, while confined cargo, for example, fluorescence, can be used for nanogel tracking. In the field of theranostics, it is pertinent that different functions have tailored conjugation behavior. Tracer and drug release is thereby tunable and the approach opens up new possibilities for designing multimodal hydrogel nanoparticles for medical applications. More diverse chemistries are still to be explored, but it has already been shown that tuning the shape (linear vs. branched) and hydrophobicity of aliphatic esters resulted in differences in release rate in solid acrylate‐based nanoparticles.^[33]^ By diversifying the ester‐conjugation in terms of hydrophobicity and steric hindrance, sequential release approaches would be possible from the same particle while maintaining the detection capabilities of using the amide‐conjugation. In short, hydrogel nanoparticles, so‐called nanogels, find their way into biomedical applications because of the possibility of loading the entire particle volume with (therapeutic and/or diagnostic) substances^[21]^, the diverse range of chemical components that can be included, as well as the ease of formation and upscaling. Introducing the multimodal approach as depicted here, these particles will become even more widely applicable.

## Conflict of interest

The authors declare the following competing financial interest(s): P.v.R also is co‐founder, scientific advisor, and shareholder of BiomACS BV, a biomedical‐oriented screening company. There are no other conflicts to declare.

## Supporting information

As a service to our authors and readers, this journal provides supporting information supplied by the authors. Such materials are peer reviewed and may be re‐organized for online delivery, but are not copy‐edited or typeset. Technical support issues arising from supporting information (other than missing files) should be addressed to the authors.

Supplementary
