# Supplementary material for: Nanogels with Selective Intracellular Reactivity for Intracellular Tracking and Delivery
Source: Chemistry. 2020 Oct 19;26(66):15084–8. doi: 10.1002/chem.202001802 (PMC7756612; doi:10.1002/chem.202001802)
Supplement: Supplementary file 1 — Supplementary [file CHEM-26-15084-s001.pdf]

Chemistry–A European Journal

Supporting Information

Nanogels with Selective Intracellular Reactivity for Intracellular Tracking and Delivery

Guangyue Zu, Olga Mergel, Laís Ribovski, Reinier Bron, Inge S. Zuhorn,* and Patrick van Rijn*[a]

PAGE

2 *METHODS AND EXPERIMENTAL DETAILS*

6 **(S1)** *SCHEMATIC ILLUSTRATION OF FLUORESCEIN RELEASE FROM FL-AC NANOGEL IN THE PRESENCE OF ESTERASE*

7 **(S2)** *^{1}H-NMR ANALYSIS OF CORE-SHELL NANOGELS*

8 **(S3)** *PARTICLE SIZE DETERMINATION AND CHARACTERISTICS*

9 **(S4)** *CYTOTOXICITY OF NANOGELS*

10 **(S5)** *CONFOCAL FLUORESCENCE IMAGING OF MCF-7 CELLS EXPOSED TO NB LABELED NANOGELS WITH CROSS-SECTIONS*

11 **(S6)** *FLUORESCENCE IMAGING OF MCF-7 CELLS EXPOSED TO A MIXTURE OF NANOGELS*

12 **(S7)** *FLUORESCENCE SPECTROSCOPY BEFORE AND AFTER CENTRIFUGATION*

13 **(S8)** *FLUORESCENCE IMAGING OF MCF-7 CELLS EXPOSED TO FLUORESCENTLY LABELED NANOGELS WITH/WITHOUT BAFILOMYCIN A1 TREATMENT*

METHODS AND EXPERIMETAL DETAILS

Materials

N-isopropylmethacrylamide (97%, NIPMAM), *N,N'*methylenebis(acrylamide) (99%, BIS), sodium dodecyl sulfate (SDS), ammonium persulfate (98%, APS), fluorescein o-acrylate (FL-Ac), 3-(acrylamido)phenylboronic acid (APBA) and methanol-d4 were purchased from Sigma-Aldrich, The Netherlands. Nile blue acrylamide (NB-AAm) was purchased from Polysciences, Inc., Germany. Methanol (≥ 99.8%) and dimethyl sulfoxide (≥ 99.0%, DMSO) were purchased from Merck, Germany. Ethanol (96%) was purchased from VWR Chemicals, The Netherlands. NIPMAM was recrystallized from hexane and dried under vacuum prior to use; All the other chemicals were used as received. Ultrapure water (18.2 MΩ, arium® 611 DI water purification system; Sartorius AG, Göttingen, Germany) was used for all synthesis reactions, purification and solution preparation.

Synthesis of Core-shell Nanogels

The core-shell nanogel with a pNIPMAM core and a pNIPMAM-co-APBA shell was synthesized through one-pot precipitation polymerization according to the literature procedure with some modifications.[1] Briefly, the polymerization was carried out in a three-necked flask equipped with a magnetic stirrer, a reflux condenser, and a nitrogen in- and outlet. To form the pNIPMAM core, monomer NIPMAM (6.65 mmol, 95 mol%), cross-linker BIS (0.35 mmol, 5 mol%), surfactant SDS (0.06 mmol) and fluorescent dye FL-Ac or NB-AAm (0.0071 mmol, 0.1 mol%) were dissolved in 48 mL water. After degassing by passing N_2 through the solution for 1 h, the solution was heated to 70 °C. The radical polymerization was initiated by injecting the degassed initiator solution of APS (0.075 mM in 2 mL water) to the reaction mixture. The initiation of polymerization was indicated by the occurrence of turbidity. The reaction solution was stirred under nitrogen atmosphere for 30 min at 70 °C. Subsequently, a comonomer mixture was added to the reaction to induce the shell synthesis. The comonomer mixture of NIPMAM (2.96 mmol, 85 mol%), BIS (0.175 mmol, 5 mol%), APBA (0.35 mmol, 10 mol%) and FL-Ac or NB-AAm (0.0035 mmol, 0.1 mol%) was dissolved in 24 mL water and degassed with N_2 over 1 h. For the preparation of the aqueous shell comonomer solution, APBA was pre-dissolved in 1 mL of methanol to increase solubility. The reaction was allowed to proceed for 6 h at 70 °C and then cooled down to room temperature and stirred overnight. The obtained nanogel was purified *via* centrifugation (Avanti J-E, Beckman Coulter GmbH, Germany) at 34400 g for 1 h, decantation of the supernatant, and redispersed in water. This procedure was repeated three times. The nanogels were further purified by dialysis (MWCO 3500 D, Fisher Scientific) against DMSO (remove unconjugated FL-Ac) or ethanol (remove unconjugated NB-AAm) for one week and following against water for one week, all the solvent was exchanged twice per day. The pure nanogels were freeze-dried for further use.

Synthesis of Dual-dye Nanogel (FL-NB nanogel)

The dual-dye core-shell nanogel with a pNIPMAM core and a pNIPMAM-co-APBA shell was synthesized through the similar procedure as above. The polymerization was carried out in a three-necked flask equipped with a magnetic stirrer, a reflux condenser, and a nitrogen in- and outlet. To form the pNIPMAM core, monomer NIPMAM (6.65 mmol, 95 mol%), cross-linker BIS (0.35 mmol, 5 mol%), surfactant SDS (0.06 mmol) were dissolved in 48 mL water. The fluorescent dye FL-Ac and NB-AAm (0.0071 mmol, 0.1 mol%) was firstly dissolve in 1 mL

DMSO and methanol, respectively, and then added to the mixture. After degassing by passing N_2 through the solution for 1 h, the solution was heated to 70 °C. The radical polymerization was initiated by injecting the degassed initiator solution of APS (0.075 mM in 2 mL water) to the reaction mixture. The reaction solution was stirred under nitrogen atmosphere for 30 min at 70 °C. Subsequently, a comonomer mixture was added to the reaction to induce the shell synthesis. The comonomer mixture of NIPMAM (2.96 mmol, 85 mol%), BIS (0.175 mmol, 5 mol%), APBA (0.35 mmol, 10 mol%) was dissolved in 24 mL water and degassed with N_2 over 1 h. For the preparation of the aqueous shell comonomer solution, APBA was pre-dissolved in 1 mL of methanol to increase solubility. The reaction was allowed to proceed for 6 h at 70 °C and then cooled down to room temperature and stirred overnight. The nanogel was purified by dialysis (MWCO 3500 D, Fisher Scientific) against DMSO (remove unconjugated FL-Ac) or ethanol (remove unconjugated NB-AAm) for one week and following against water for one week, all the solvent was exchanged twice per day. The pure nanogels were freeze-dried for further use.

In order to confirm the presence of both dyes within the nanogel, the nanogels were imaged using fluorescence microscopy and identifying the colocalization of both emission signals. The separate images and colocalization image are shown below.

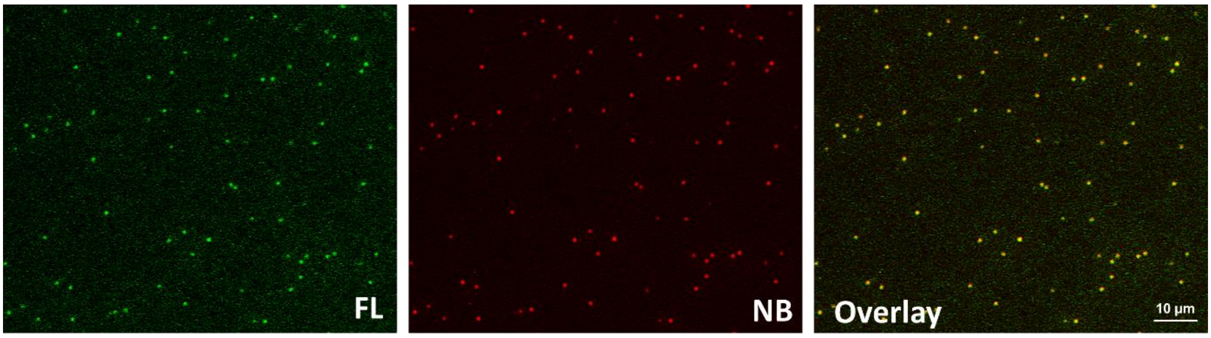

Nuclear Magnetic Resonance Spectra

Nuclear Magnetic Resonance spectra were measured with a Varian Mercury-400 NMR spectrometer (400 MHz). All spectra were measured at room temperature. Methanol-d4 was used as solvent and the nanogel concentration was 10 mg/mL. The proton signal of residual methanol-d4 was used as reference.

Transmission Electron Microscopy

The nanogels were observed using a Philips CM120 Microscope coupled to a 4k CCD camera using an acceleration voltage of 120 kV. All the samples were negatively stained with supersaturated uranyl acetate and drop casted on carbon film coated Cu grids.

Temperature Dependent Dynamic Light Scattering

The hydrodynamic diameters (D_h) and polydispersities of the nanogels were determined by Dynamic Light Scattering (DLS). The measurements were performed using a Malvern ZetaSizer ZS ZEN3600 machine (Malvern Instruments, U.K.) equipped with a temperature controller. The scattering detector positioned at a fixed scattering angle of 173°. The concentrations of nanogel dispersions were around 0.1 mg/mL. Hydrodynamic diameters were calculated from diffusion coefficient using the Stokes−Einstein equation. The polydispersity

index is given by the cumulant analysis method. All correlogram analyses were performed with software supplied by the manufacturer. Temperature dependent measurements were performed in a range of 20 ~ 60 °C with 2 °C intervals. Before data collection of each temperature, the sample was allowed to equilibrate 2 min at the proper temperature. Each data point is an average of three successive hydrodynamic diameters measurements, which themselves consist of 11-15 measurements with approx. 15 s integration time.

Cell Culture

Human breast cancer cell line Michigan Cancer Foundation-7 cells (MCF-7 cells) and Mouse fibroblast cell line (L929 cells) were cultured in Dulbecco's Modified Eagle Medium (DMEM, high glucose, Gibco) and Minimum Essential Medium (MEM, Gibco), respectively. Both culture mediums were supplemented with 10% fetal bovine serum (Gibco) and 100 units/mL of streptomycin and penicillin (Gibco). The cells were maintained at 37 °C in a humidified atmosphere of 5% CO_2 in air.

Confocal Laser Scanning Microscopy

The cellular uptake and intracellular behavior of nanogels were determined by confocal laser scanning microscopy (CLSM). MCF-7 cells were seeded in 6-well plates at ~200000 cells per well in 2 mL DMEM medium and incubated for 24 h. After removing the culture medium, the cells were treated with FL-Ac nanogel and/or NB-AAm nanogel and FL-NB nanogel at a final concentration of 0.5 mg/mL in Hank's Balanced Salt Solution (HBSS, Gibco) for 2 h. Cells without treatment were used as control. Then the HBSS was removed and cells were washed with PBS three times. To show location of nuclei, the cells were fixed with 3.7% paraformaldehyde (Sigma-Aldrich) in PBS for 30 min at room temperature, and the cell nuclei were stained with 4',6-diamidino-2-phenylindole (DAPI, Sigma-Aldrich). Thereafter, CLSM images of cells were obtained with a Leica TCS SP2 Confocal microscope. Background signal was avoided by adjusting the settings based on control cells.

To investigate the effect of Bafilomycin A1 on endocytic transport of nanogels, MCF-7 cells were incubated in serum-free DMEM medium for 2 h at 37°C in the absence or presence of 200 nM Bafilomycin A1 (Sigma-Aldrich) after which nanogels were added and cells were incubated for another 2 h .

Fluorescence Spectroscopy

MCF-7 cells were cultured to approximately 80% confluence and harvested. After washing with PBS, the cells were lysed by adding water and treated with ultrasound. Afterwards, the FL-NB nanogel and NB-AAm nanogel were incubated with or without the cell lysate in HBSS and water at a final nanogel concentration of 0.5 mg/mL at 37 °C. The pH of all nanogel dispersions was around 7. After 2 h incubation, the supernatant was collected *via* ultracentrifugation (Sorvall Discovery 90SE, Cambridge Scientific Products, USA) of the suspensions at 23700 g for 1 h for fluorescence measurement. The fluorescence emission spectrum was recorded at 25°C using a Synergy H1 Multi-Mode Reader at excitation wavelength of 488 nm for FL-Ac and 630 nm for NB-AAm. Ultrapure water or HBSS buffer was measured as a reference.

Cytotoxicity Assay

2,3-bis(2-methoxy-4-nitro-5-sulfophenyl)-2H-tetrazolium-5-carboxanilide (XTT) assay (AppliChem, A8088) was performed (n=3) to evaluate the cytotoxicity of nanogels. L929 cells were seeded into 96-well plates at a density of 8000 cells per well in 100 μL of MEM complete medium. Wells containing 100 μL of medium alone were included as blank absorbance readings. After 24 h of incubation, the medium was replaced with 100 μL of fresh medium containing nanogels at various concentrations, and incubated for another 24 h. Cells without treatment were used as control. Afterwards, 50 μL of XTT solution was added to each well and the cells were then incubated for another 5 h. After gently shaking, 100 μL of the supernatant was transferred to a 96-well plate and the absorbance at 490 nm was measured with FluoStar Optima Plate reader. To avoid non-specific readings, the absorbance at 690 nm was measured and subtracted from 490 nm measurement. The cell viability was determined by the formula:

$$\text{Relative cell viability (\%)} = 100 \times (\Delta Abs_{sample} - \Delta Abs_{blank}) / (\Delta Abs_{control} - \Delta Abs_{blank})$$

$$\Delta Abs = Abs(490\ nm) - Abs(690\ nm)$$

S1 SCHEMATIC ILLUSTRATION OF FLUORESCEIN RELEASE FROM FL-AC NANOGEL IN THE PRESENCE OF ESTERASE

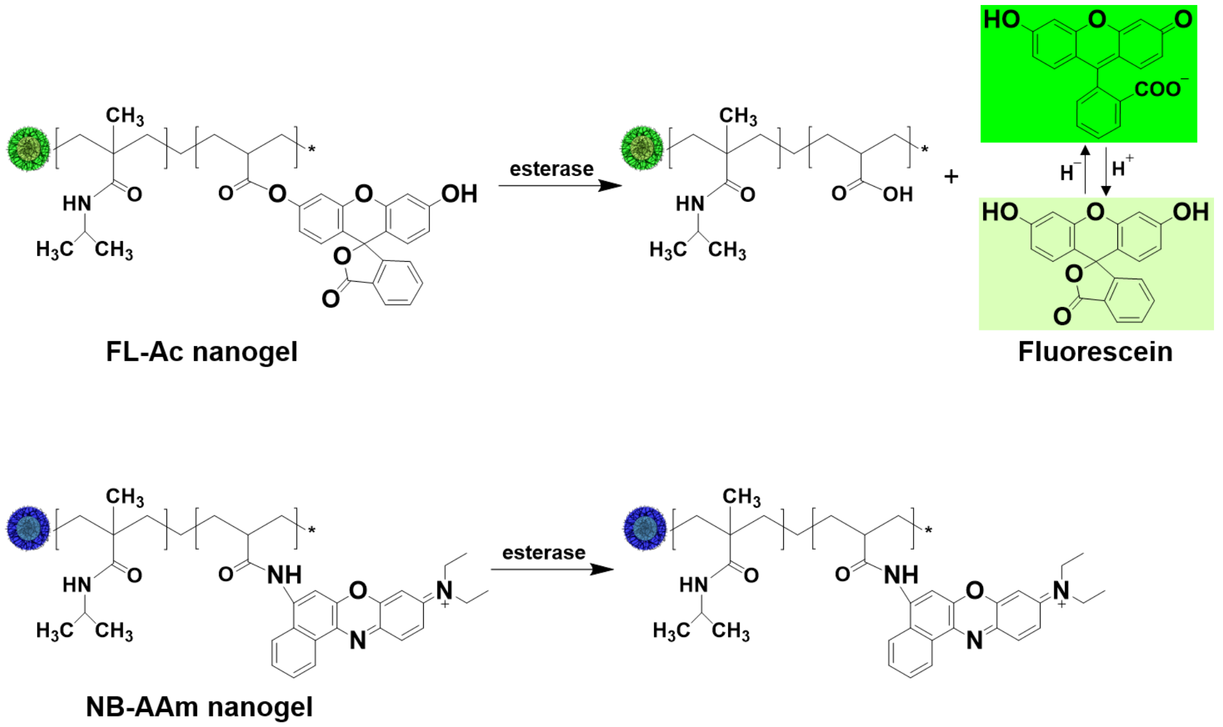

Figure S1. Schematic illustration of fluorescein release from FL-Ac nanogel in the presence of esterase, while the NB-AAm nanogel is stable.

S2 ^{1}H-NMR ANALYSIS OF CORE-SHELL NANOGELS

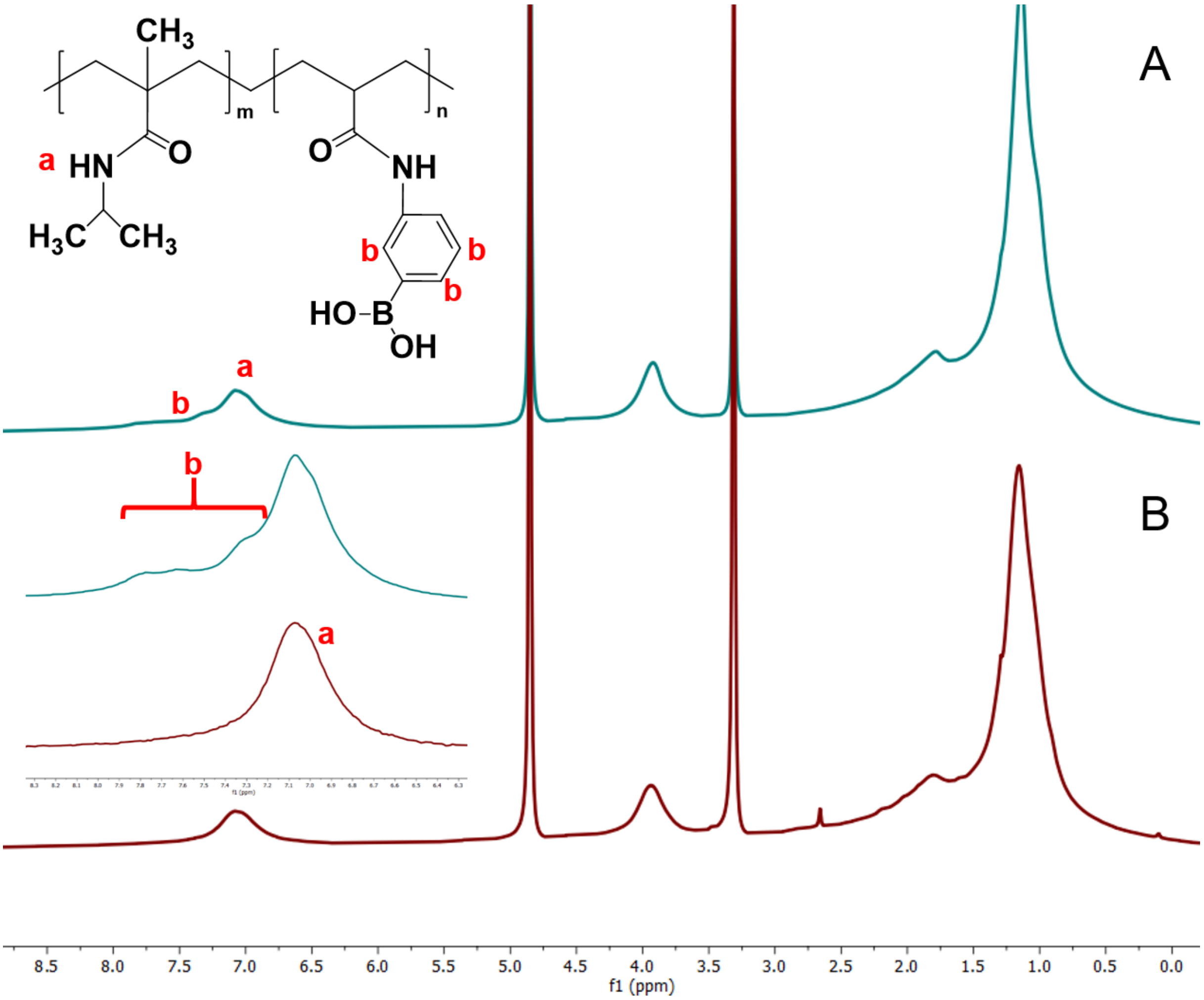

Figure S1. ^{1}H-NMR spectra of pNIPMAM-APBA-FL-Ac nanogel (A) and pNIPMAM-FL-Ac nanogel (B). Inserted figure is the magnification of the part of 6.40-8.30 of chemical shift.

S3 PARTICLE SIZE DETERMINATION AND CHARACTERISTICS

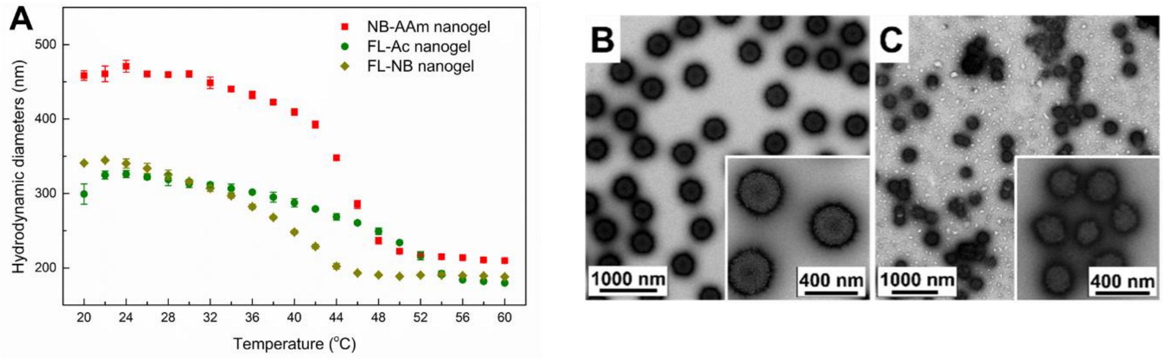

Figure S3. Hydrodynamic diameter (D_h) against temperature FL-Ac, NB-AAm and FL-NB-nanogels obtained by Dynamic Light Scattering (A). Transmission electron microscopy images of NB-AAm nanogel (B) and FL-Ac nanogel (C).

S4 CYTOTOXICITY OF NANOGELS

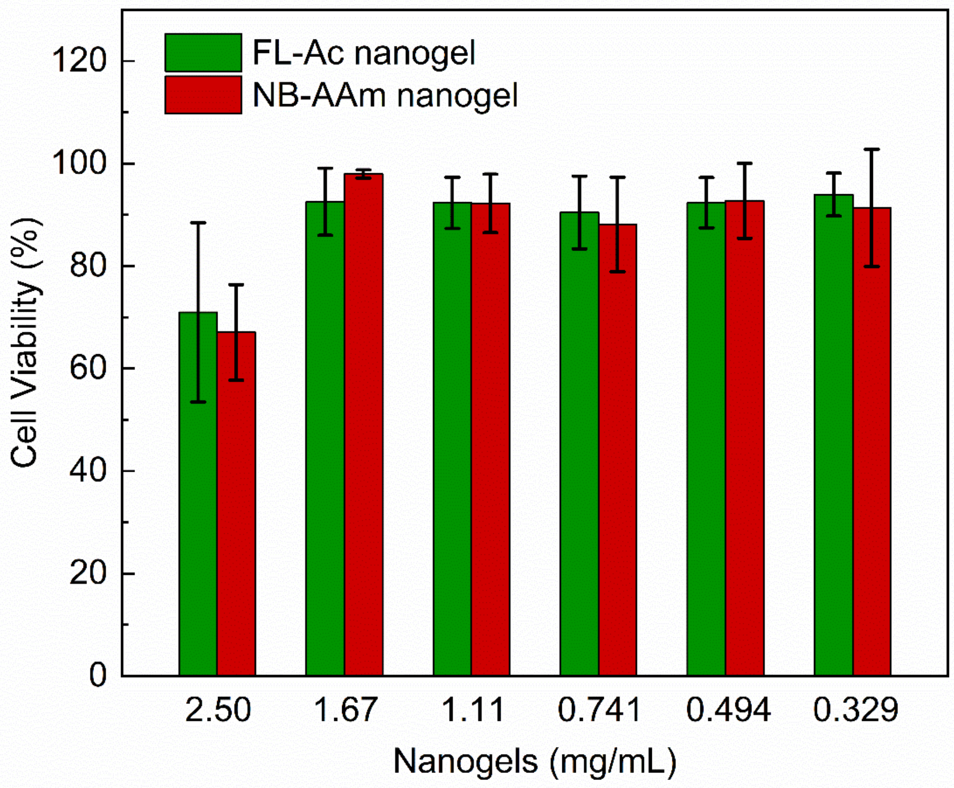

Figure S4. Cell viability of FL-Ac nanogel and NB-AAm nanogel. L929 fibroblasts cells were treated with nanogels for 24 h at 37°C and the cytotoxicity was determined by XTT assay.

S5 CONFOCAL FLUORESCENCE IMAGING OF MCF-7 CELLS EXPOSED TO NB LABELED NANOGELS WITH CROSS-SECTIONS

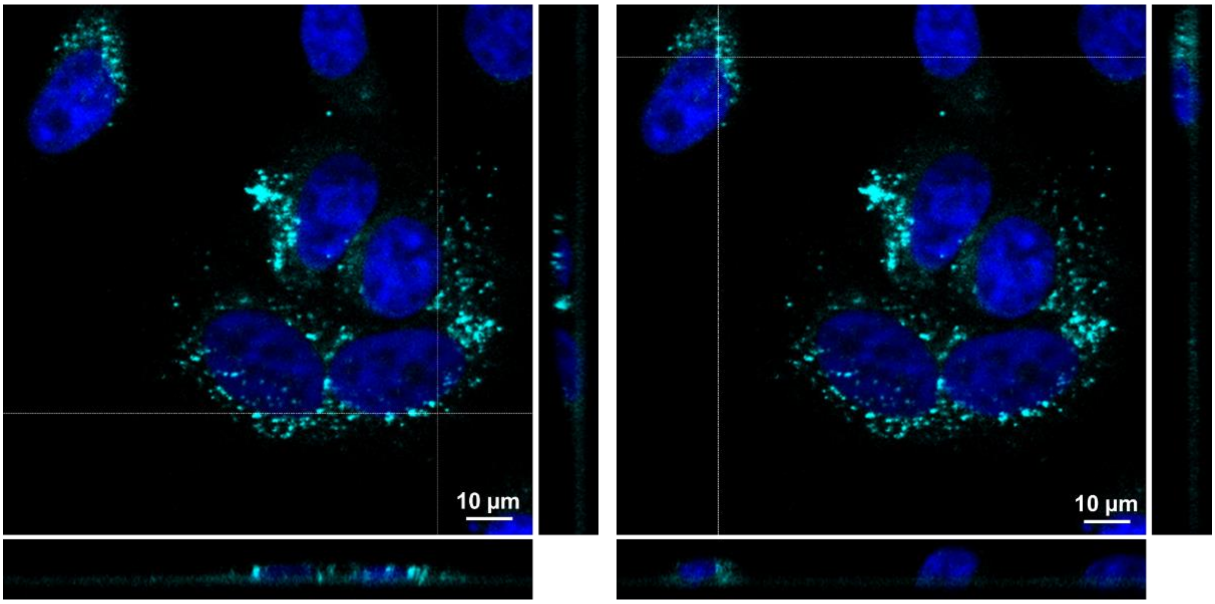

Figure S5. Fluorescence confocal microscopy of MCF-7 breast cancer cells incubated with NB-AAm nanogels for 2 h at 37°C. In addition to the 2D distribution, the cross-section of the cells obtained by imaging various focal planes in the z-direction further substantiates the internalization of the nanogels inside the cells.

S6 FLUORESCENCE IMAGING OF MCF-7 CELLS EXPOSED TO A MIXTURE OF FLUORESCENTLY LABELED NANOGELS

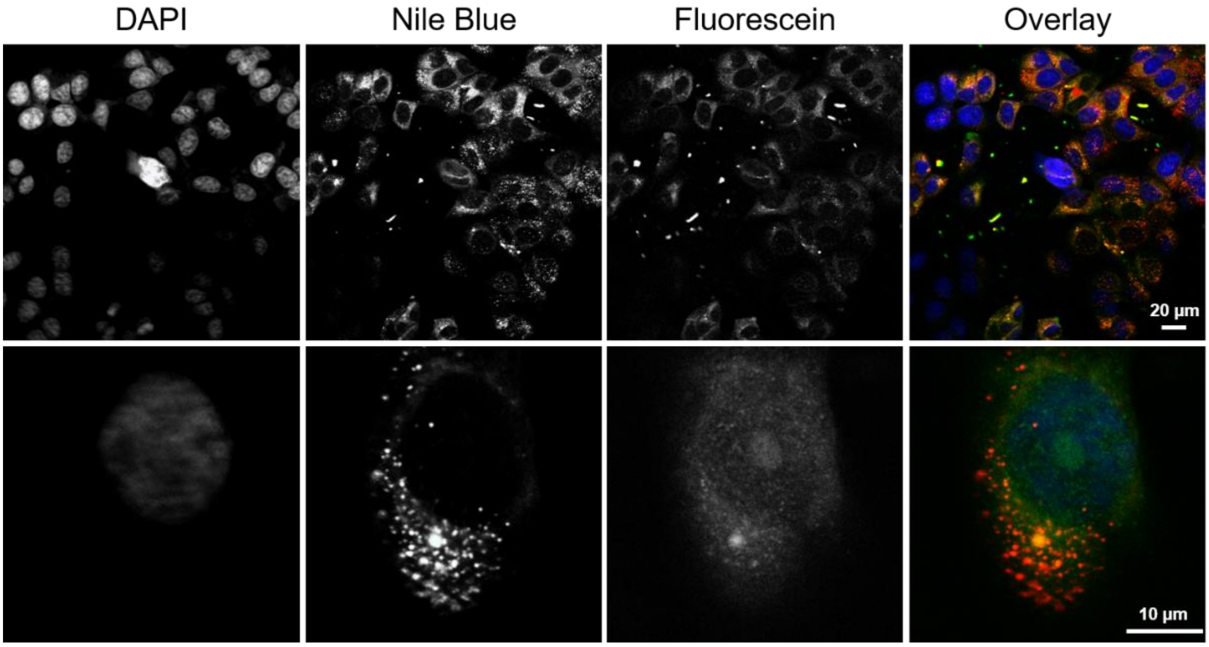

Figure S6. Fluorescence confocal microscopy of MCF-7 breast cancer cells co-incubated with a mixture of NB-AAm and FL-Ac nanogels for 2 h at 37°C. Top row: Nile Blue and Fluorescein fluorescence signals show overlap in MCF-7 cells co-incubated with the two types of nanogels. Bottom row: High magnification images further demonstrate that the amide-conjugated Nile Blue (red) displays a dotted pattern within the cell cytosol reminiscent of an endosomal distribution, while the ester-conjugated Fluorescein (green) shows more fuzzy dots that are more widely distributed over the entire cell. DAPI (blue) is used for staining the nucleus.

S7 FLUORESCENCE SPECTROSCOPY BEFORE AND AFTER CENTRIFUGATION

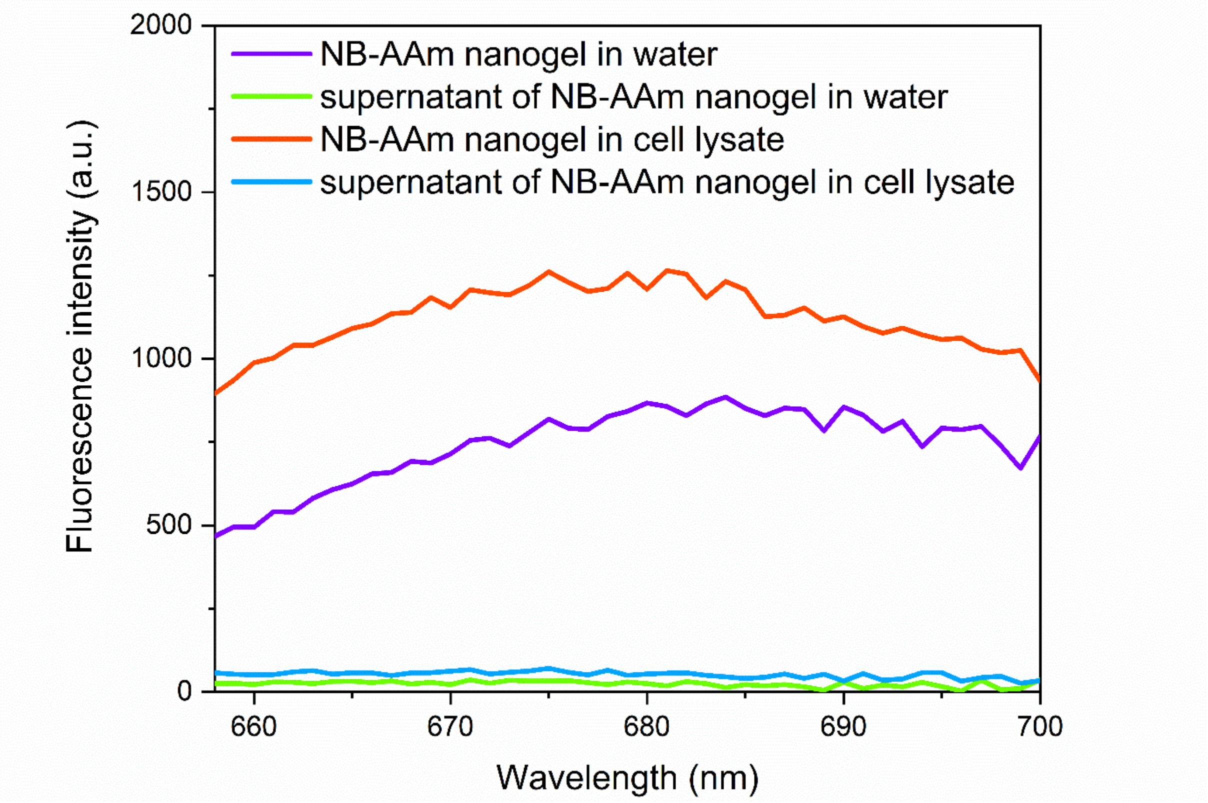

Figure S7. Fluorescence spectroscopy of NB-AAm nanogel and supernatant following nanogel exposure to water or cell lysate. After exposure of nanogels to water or cell lysate, the nanogels were pelleted through ultracentrifugation. Fluorescence of the nanogel pellet and the supernatant was measured. Note the absence of fluorescence in the supernatant following nanogel exposure to water and cell lysate, indicating successful pelleting of nanogels by ultracentrifugation and absence of dye release from NB-AAm nanogels in the presence of cell lysate, respectively.

S8 FLUORESCENCE IMAGING OF MCF-7 CELLS EXPOSED TO FLUORESCENTLY LABELED NANOGELS WITH/WITHOUT BAFILOMYCIN A1 TREATMENT

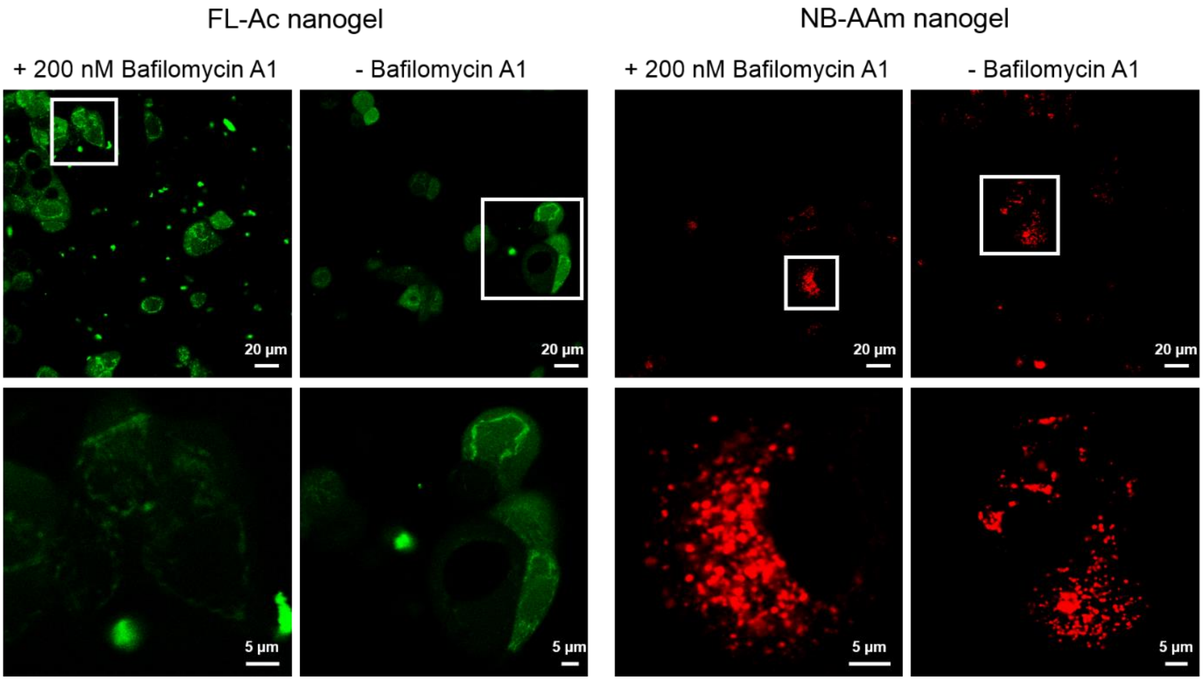

Figure S8. Influence of bafilomycin A1 on endocytic transport of FL-Ac and NB-AAm nanogels as analyzed by fluorescence confocal microscopy. MCF-7 cells were pre-incubated without or with bafilomycin A1 (200 nM) for 2 h at 37°C followed by incubation with NB-AAm or FL-Ac nanogels for another 2 h at 37°C. Top row: FL-Ac and NB-AAm nanogels display the same distribution in cells treated with and without bafilomycin A1. NB-AAm nanogel displays a dotted pattern within the cell cytosol reminiscent of an endosomal distribution, while Fl-Ac nanogel displays fuzzy dots more widely distributed over the entire cell and reticular structures next to the nucleus. Bottom row: High magnification images of boxed areas in top row.

References

[1] V. Lapeyre, C. Ancla, B. Catargi, V. Ravaine, *J. Colloid Interface Sci.* **2008**, *327*, 316–323.
